# Vancomycin-intermediate livestock-associated methicillin-resistant *Staphylococcus aureus* ST398/t9538 from swine in Brazil

**DOI:** 10.1590/0074-02760160276

**Published:** 2016-10

**Authors:** Luisa Z Moreno, Mauricio C Dutra, Marina Moreno, Thais SP Ferreira, Givago FR da Silva, Carlos EC Matajira, Ana Paula S Silva, Andrea M Moreno

**Affiliations:** Universidade de São Paulo, Faculdade de Medicina Veterinária e Zootecnia, Laboratório de Sanidade Suína, São Paulo, SP, Brasil

**Keywords:** LA-MRSA, vancomycin resistance, swine

## Abstract

Livestock-associated methicillin-resistant *Staphylococcus aureus* (LA-MRSA) has been mainly related with pig farming, in Europe and North America, with the ST398 as the most commonly identified type of LA-MRSA. Here we present the draft genome of the first vancomycin-intermediate MRSA ST398/t9538 isolated from a swine presenting exudative epidermitis in Brazil.

Methicillin-resistant *Staphylococcus aureus* (MRSA) animal infection has been reported since the 1970s and is now referred to as livestock-associated MRSA (LA-MRSA). It has only been since 2005, with the studies of MRSA pig-associated strains from sequence type 398, that LA-MRSA started to have greater importance to the medical-scientific community (Leonard & Markey 2008). In Europe and North America, the ST398 remains the most commonly identified type of LA-MRSA (Smith 2015); nevertheless, clinical infections by LA-MRSA ST398 are still rare.

In South America, LA-MRSA ST398 has only been associated with porcine carriage in Peru (Arriola et al. 2011) and milk contamination in Brazil (Silva et al. 2014). The epidemiology and public health impact of LA-MRSA in South America remains poorly addressed. Here we present the isolation, phenotypic and genomic characterisation of MRSA ST398/t9538 from a swine presenting exudative epidermitis in Brazil.

The strain SA7112 was isolated in 2012, from a skin swab collected from a 45-day-old swine presenting skin exfoliation with sebaceous exudation and crust formation in Rio Grande do Sul state, Brazil. The skin swab was plated on sheep blood agar (5%) and incubated for 24 h at 37ºC. The hemolytic white colonies were identified as *S. aureus* by polymerase chain reaction (PCR) as described by Kearns et al. (1999). A single colony was used for: antimicrobial susceptibility profiling, research of *mec*A gene by PCR (Kearns et al. 1999) and further genome sequencing.

The minimal inhibitory concentration (MIC) was determined by broth microdilution technique (CLSI 2013) using GPALL1F and BOPO6F Sensititre^®^ Standard Susceptibility MIC Plates (TREK Diagnostic Systems/Thermo Fisher Scientific). *S. aureus* ATCC 29213 was used as quality control. The interpretative breakpoints were obtained in the supplements VET01-S2 (CLSI 2013) and M100-S24 (CLSI 2014). SA7112 was resistant to the tested β-lactams with oxacillin MIC > 4.0 µg/mL confirming the MRSA phenotype. Interestingly, the isolate also presented a vancomycin-intermediate phenotype. SA7112 presented an alarming multirresistant profile with resistance to aminoglycosides, macrolides, tetracyclines, sulfonamides, fluoroquinolones, phenicols, clindamycin, quinupristin/dalfopristin and tiamulin (Table I).

**TABLE I:** Minimum inhibitory (MIC) concentration values, antimicrobials testing range and breakpoints applied for SA7112 antimicrobial profiling

Antibiotics	Testing Range (µg/mL)	MIC (µg/mL)	MIC Breakpoints		

			Susceptible	Intermediate	Resistant
Penicillin	0.12 - 8.0	> 8.0	≤ 0.12	-	≥ 0.25
Oxacillin	0.25 - 4	> 4.0	≤ 2.0	-	≥ 4.0
Ampicillin	0.12 - 16	16.0	≤ 0.25	-	≥ 0.5
Ceftiofur	≤ 0.25 - 2.0	> 8.0	≤ 2.0	4.0	≥ 8.0
Vancomycin	0.25 - 16	8.0	≤ 4.0	8.0 - 16.0	≥ 32.0
Spectinomycin	8.0 - 64.0	> 64.0	≤ 32.0	-	≥ 64.0
Streptomycin	1000	> 1000	-	-	> 1000
Erythromycin	0.25 - 4.0	> 4.0	≤ 0.5	1.0 - 4.0	≥ 8.0
Tylosin	0.5 - 32.0	> 32.0	≤ 1.0	2.0 - 4.0	˃ 4.0
Tilmicosin	4.0 - 64.0	> 64.0	≤ 16.0	-	≥ 32.0
Tulathromycin	1.0 - 64.0	> 64.0	≤ 16.0	32.0	≥ 64.0
Tetracycline	2.0 - 16.0	> 16.0	≤ 4.0	8.0	≥ 16.0
Chlortetracycline	0.5 - 8.0	> 8.0	≤ 0.5	1.0	≥ 2.0
Oxytetracycline	0.5 - 8.0	> 8.0	≤ 0.5	1.0	≥ 2.0
Moxifloxacin*	0.25 - 4.0	> 4.0	≤ 0.5	1.0	≥ 2.0
Levofloxacin*	0.25 - 4.0	> 4.0	≤ 1.0	2.0	≥ 4.0
Ciprofloxacin*	1 - 2	> 2.0	≤ 1.0	2.0	≥ 4.0
Danofloxacin	0.12 - 1.0	> 1.0	≤ 0.25	-	-
Enrofloxacin	0.12 - 2.0	> 2.0	≤ 0.5	1.0	≥ 2.0
Clindamycin	0.25 - 16.0	> 16.0	≤ 0.5	1.0 - 2.0	≥ 4.0
Chloramphenicol	2 - 16	> 16.0	≤ 8.0	16.0	≥ 32.0
Florfenicol	0.25 - 8.0	> 8.0	≤ 2.0	4.0	≥ 8.0
Quinuptn/Dalfoptn*	0.5 - 4	> 4.0	≤ 1.0	2.0	≥ 4.0
Trimetop/Sulfametx	0.5/9.5 - 4/76	> 4/76	≤ 2/38	-	≥ 4/76
Sulfadimethoxine	256.0	> 256.0	≤ 256.0	-	>256.0
Tiamulin	0.5 - 32.0	> 32.0	≤ 16.0	-	≥ 32.0

*: applied breakpoints from CLSI document M100-S24 (CLSI 2014).

Whole genome sequencing was performed through Illumina^®^ Miseq platform with paired-end library. The *de novo* assembly was performed with CLC Main Workbench 7.5.1 (CLC Bio, Denmark) and Geneious 8.0.5 (Biomatters Ltd, Auckland, New Zealand) and resulted in 22 scaffolds with an N_50_ of 466,156. Mapping and ordering of obtained scaffolds with reference strain S0385, an MRSA ST398 (NC_017333), was performed with CLC Microbial Genomics Module (CLC Bio, Denmark) and Mauve multiple genome aligner (Darling et al. 2010) and demonstrated the existence of two plasmids pSA7112-1 (KX011076) and pSA7112-2 (KX011077) (Table II). The SA7112 draft genome (LNTF00000000.1) comprises ~2.8 Mbp, with an overall G+C content of 32.89%. Automatic genome annotation was performed with NCBI Prokaryotic Genome Annotation Pipeline.

**TABLE II:** Assembly statistics and basic annotation features of Brazilian livestock-associated methicillin-resistant *Staphylococcus aureus* SA7112 strain chromosome and plasmids

	Chromosome	pSA7112-1	pSA7112-2
Length	2,805,326	2,366	44,430
Scaffolds	20	1	1
N_50_	466,156	-	-
CG%	32.89	31.07	32.80
CDS	2,660	2	30
rRNAs	18	-	-
tRNAs	59	-	-

Chromosomal analysis enabled SA7112 typing as SCCmec V(5C2&5) subtype c, *spa* t9538 and ST398. The ST398 has only been reported in one human methicillin- susceptible *S. aureus* (MSSA) Brazilian strain (Gales et al. 2015) that was further typed as *spa* t034. Even though the identified *spa* t9538 has not been associated with LA-MRSA, it is closely related to t034 that is characterised as a livestock-associated *spa* type. While this is the first report of LA-MRSA ST398 carrying a SCCmec type V(5C2&5) subtype c in Brazil, it has already been established as the predominant LA-MRSA in Europe (Li et al. 2011).

The SA7112 genome presents the genes encoding aureolysin (*aur*), beta-hemolysin (*hlb*) and staphylococcal gamma-hemolysins (*hlgA*, *hlgB* and *hlgC*), which are related to bacteria escaping from the host immune system. The *lukM-lukF-PV* (Panton-Valentine bi-component leukotoxin) and the exfoliative toxin genes were not identified. With regard to the resistance profile, only the *fexA*, *norA*, *tetM* and *tetK* resistance genes were detected in SA7112 chromosome while both identified plasmids appear to be mostly responsible for the SA7112 resistance phenotype.

The pSA7112-1 corresponds to an *ermC* plasmid, commonly found in *S. aureus* isolates, while pSA7112-2 comprises a multidrug-resistant plasmid harboring *aadE*, *aadD*, *tetL* and *dfrK* genes. The absence of *van* genes and the multifactorial aspect of vancomycin intermediate resistance indicate the possibility of alterations in the cell wall thickness as responsible for the observed phenotype (Hiramatsu et al. 2001).

This is the first report of vancomycin-intermediate LA-MRSA ST398/t9538 in Brazil. It highlights the public health risk for dissemination such a multidrug-resistant pathogen, not only to slaughterhouse workers and pig farmers, but also to the community due to the contamination risk of retail pork. There already exists a report of MSSA ST398 in a Brazilian hospital with multi-resistant phenotype (Gales et al. 2015). This indicates that the livestock-associated clone (ST398) is already present in Brazilian territory and is underestimated due to the lack of surveillance studies. The identification of vancomycin-intermediate LA-MRSA confirms the existing underrated high risk to public health and, therefore, the necessity to enhance LA-MRSA epidemiological studies in South America.
